# Supplementary material for: Texture and semantic integrated small objects detection in foggy scenes
Source: PLoS One. 2022 Aug 18;17(8):e0270356. doi: 10.1371/journal.pone.0270356 (PMC9387851; doi:10.1371/journal.pone.0270356)
Supplement: S1 Dataset — (DOCX) [file pone.0270356.s001.docx]

3. We note that your Data Availability Statement reads, "A large number of experiments were carried out on two available large-scale data sets, i.e. "Cityscape to Foggy"(Hahner. M, Dai. D, Sakaridis. C, Zaech. J and Gool. L. V. Semantic Understanding of Foggy Scenes with Purely Synthetic Data[C]. IEEE Intelligent Transportation Systems Conference (ITSC), Auckland, New Zealand, 2019:3675-3681.) and "CoCo"(Lin. T, Maire. M, Belongie. S, Hays. J, Perona. P, Ramanan. D, Dollar. P and Zitnick. C. Microsoft COCO: Common Objects in Context[C]. European Conference on Computer Vision (ECCV), Zurich, Switzerland, 2014: 740-755.)." Could you please provide DOIs to each of the data sets as well? We will update your Data Availability Statement on your behalf with the information you provide.

(1) "Cityscape to Foggy"(Hahner. M, Dai. D, Sakaridis. C, Zaech. J and Gool. L. V. Semantic Understanding of Foggy Scenes with Purely Synthetic Data[C]. IEEE Intelligent Transportation Systems Conference (ITSC), Auckland, New Zealand, 2019:3675-3681.)

Cityscape to Foggy  Datasets：<https://people.ee.ethz.ch/~csakarid/SFSU_synthetic/>

(2) "CoCo"(Lin. T, Maire. M, Belongie. S, Hays. J, Perona. P, Ramanan. D, Dollar. P and Zitnick. C. Microsoft COCO: Common Objects in Context[C]. European Conference on Computer Vision (ECCV), Zurich, Switzerland, 2014: 740-755.)

CoCo Datasets： https://cocodataset.org/#home
