# Supplementary material for: Texture and semantic integrated small objects detection in foggy scenes
Source: PLoS One. 2022 Aug 18;17(8):e0270356. doi: 10.1371/journal.pone.0270356 (PMC9387851; doi:10.1371/journal.pone.0270356)
Supplement: Attachment — Submitted filename: renamed_39106.pdf [file pone.0270356.s003.pdf]

Review comments：

This paper proposes a knowledge-guided and information-rich small target detection algorithm in foggy scenes. The algorithm focuses on extracting discriminative features unaffected by the environment, and obtaining texture information and high-level semantic information of small targets. Specifically, considering the adverse impact of foggy images on recognition performance, a knowledge guidance module is designed, and the discriminative features extracted from clear images by the model are used to guide the network to learn foggy images. Second, the features of high-resolution images and low-resolution images are extracted, and the adversarial learning method is adopted to train the model to give the network the ability to obtain the texture information of small targets from the low resolution images. Finally, an attention mechanism is constructed between feature maps of the same scale and different scales to further enrich the high-level semantic information of small targets. A large number of experiments have been conducted on data sets such as “Cityscape to Foggy” and “CoCo”. The mean prediction accuracy (mAP) has reached 42.8% on “Cityscape to Foggy”, and 33.3 % on “CoCo”, which fully proves the effectiveness and superiority of the proposed method.

In general, this paper is well written, but I have to point out that there are still some grammar and word mistake. Moreover, the author should also solve the following limitations:

1. The author should polish the language of the paper.

2. It is suggested that the paper should be revised in strict accordance with the required format. For example: the position marked above the author's name and affiliations; each paragraph is indented and so on.

3. The recommended font must meet the requirements for the template.

4. The main motivation and contribution should be clearly highlighted in the introduction section.

5. The conclusion of the article is too simple, it should be described more fully to illustrate the feasibility and advantages of the method in this article.

6. It is recommended that the requirements of references and templates be strictly unified.

7. The naming format of the diagram is checked against the template.

8. As shown in Figure 2, the description of the algorithm should not be in the form of screenshots. It is recommended to replace it with Table.

9. The arrow flow of each diagram is recommended to be described in colloquial language for easy reading.

10. It is recommended to specify the color of the arrow and the meaning of the dotted and solid

lines in each diagram.

11. The content and conclusion of the study are too simple, so I suggest to enrich the content of you work.

In order to make this article more fluent and easier to understand, I suggest the authors consider about those above eleven problems.
