# Supplementary material for: Texture and semantic integrated small objects detection in foggy scenes
Source: PLoS One. 2022 Aug 18;17(8):e0270356. doi: 10.1371/journal.pone.0270356 (PMC9387851; doi:10.1371/journal.pone.0270356)
Supplement: Attachment — Submitted filename: Review Report.docx [file pone.0270356.s002.docx]

Dear authors,

The manuscript titled “Knowledge-guided and information-rich small target detection in foggy scenes” combines texture information and semantic information to detect small target in the foggy images. The work is interesting and the result support the conclusion well. However there are some flaws necessary to be polished to improve the quality of this manuscript. The novelty of the research is not outstandingly presented; the English language should be improved either. My detailed comments are as below

**Point 1:** In the title, the short-phrase ‘Knowledge-guided and information-rich small target detection in foggy scenes 'could be rewritten making it be more understandable. Here knowledge and information are abstracted from the image because of fog or are used in a method/process?

**Point 2:** In the abstract, the conclusion of ‘the existing small target detection algorithms ignore the texture information’ is not very precise. U can correct it with ‘some of the existing small target detection algorithms’.

**Point 3:** The abstract should be rewritten carefully; then I could find how your experiment arranged logically.

**Point 4:** Self-attention mechanism is applied in semantic information enhancement, and it is a very important part in this manuscript so I think it should be added in keywords.

**Point 5:** In lines 134-136, I recommend to add citations supporting the statement of self-attention. Because it is not the conclusion of the article itself, so it should be expressed clearly.

**Point 6:** According to the foggy images, have u used any method to defog these images? If u have had some experiments, please show the results in the manuscript. If not, u can defogging these images, and they can be input to ur proposed algorithm. If u don’t think it necessary, please tell me why.

Above are some of my concerns with the manuscript. I recommend examining carefully the language and the logic in the presentation of the manuscript before submission.
