# Supplementary material for: Texture and semantic integrated small objects detection in foggy scenes
Source: PLoS One. 2022 Aug 18;17(8):e0270356. doi: 10.1371/journal.pone.0270356 (PMC9387851; doi:10.1371/journal.pone.0270356)
Supplement: Attachment — Submitted filename: Response to reviewers.docx [file pone.0270356.s004.docx]

《PLOS ONE》Response to reviewers

Thank you for allowing a resubmission of our manuscript, with an opportunity to address the reviewers’ comments.

**Journal Requirements:**

**Case1:** Please ensure that your manuscript meets PLOS ONE's style requirements, including those for file naming.

**Response:** We revised this manuscript accordance with the PLOS ONE's style requirements.

**Case2:** Please ensure that you refer to Figure 6 in your text as, if accepted, production will need this reference to link the reader to the figure.

**Response:** We revised Figure 6 according to the algorithm’ style requirements, such as algorithm 1.

**Case3:** We note that Figures 1 and 2 in your submission contain [map/satellite] images which may be copyrighted.

**Response:** We revised them. Such as Figures 1 and 2. The pictures in Figure 1 and Figure 2 are taken by us, and there is no copyright problem.

**Reviewer #1:**

**Case1:** The manuscript titled “Knowledge-guided and information-rich small target detection in foggy scenes” combines texture information and semantic information to detect small target in the foggy images. The work is interesting and the result support the conclusion well. However there are some flaws necessary to be polished to improve the quality of this manuscript. The novelty of the research is not outstandingly presented; the English language should be improved either.

**Response:** The quality of this manuscript has been improved, the existing English language problems have been improved, and the novelty of the research is also improved.

**Reviewer #2:**

**Case1:** The author should polish the language of the paper.

**Response:** The existing English language problems have been improved.

**Case2:** It is suggested that the paper should be revised in strict accordance with the required format. For example: the position marked above the author's name and affiliations; each paragraph is indented and so on.

**Response:** We revised this manuscript accordance with the required format.

**Case3:** The recommended font must meet the requirements for the template.

**Response:** I rearranged the typesetting according to the PLOS ONE's Latex template.

**Case4:** The main motivation and contribution should be clearly highlighted in the introduction section.

**Response:** We revised this manuscript in the introduction section.

**Case5:** The conclusion of the article is too simple, it should be described more fully to illustrate the feasibility and advantages of the method in this article.

**Response:** We supplemented the conclusion of the article.

**Case6:** It is recommended that the requirements of references and templates be strictly unified.

**Response:** We revised it according to the requirements of references and templates.

**Case7:** The naming format of the diagram is checked against the template.

**Response:** We revised it.

**Case8:** As shown in Figure 2, the description of the algorithm should not be in the form of screenshots. It is recommended to replace it with Table.

**Response:** We revised it according to the algorithm’ style requirements, such as algorithm 1.

**Case9:** The arrow flow of each diagram is recommended to be described in colloquial language for easy reading.

**Response:** Thank you for your suggestions. In the revision manuscript, we have added arrow examples to facilitate readers' reading.

**Case10:** It is recommended to specify the color of the arrow and the meaning of the dotted and solid lines in each diagram.

**Response:** Thank you for your suggestions. In the revision manuscript, we have added arrows of different colors and the meaning of points.

**Case11:** The content and conclusion of the study are too simple, so I suggest to enrich the content of you work.

**Response:** Thank you very much for your suggestions. We added the semantic location information fusion attention module to make the deep features extracted by the feature extractor have stronger location representation, so as to assist the semantic information enhancement module and enrich the location information of large-scale features.

**Reviewer #3:**

**Case1:** Consider improving and adding a more suitable knowledge distillation model structure for application. The part of the ablation experiment is just the split and combination of each module, and more adequate experiments can be considered.

**Response:** Thank you for your suggestions. In the revision manuscript, we modified the model and supplemented the ablation experiments combined with different modules.

**Case2:** Two feature extractors E are used to obtain Eh. Is it redundant? Can it be replaced by a general feature extractor, with two different resolutions as input, so that E and D confront each other;

**Response:** Thank you for your question. Our idea is to enable feature extractor E to propose high-resolution features for low-resolution images. If the weight is shared, it is difficult to extract good high-resolution features for high-resolution images, so it is difficult to guide feature extractor E to extract high-resolution features for low-resolution images. Therefore, we set up two feature extractors Eh and E to ensure better quality of extracted high-resolution features.

**Case3:** The algorithm flow is not clear enough, the algorithm format is wrong, it should not be a screenshot;

**Response:** We revised it according to the algorithm’ style requirements, such as algorithm 1.

**Case4:** English translation has grammatical errors or has not been investigated, such as target detection.

**Response:** We have modified the English translation grammatical errors and the improper English expressions in this paper.

**Case5:** There is a lack of detailed process description below the paper frame diagram.

**Response:** Thank you very much for your suggestions. In the revision manuscript, we added a detailed process description under the framework chart of the paper.

**Reviewer #4:**

**Point 1:** In the title, the short-phrase ‘Knowledge-guided and information-rich small target detection in foggy scenes 'could be rewritten making it be more understandable. Here knowledge and information are abstracted from the image because of fog or are used in a method/process?

**Response:** We modified the title as “Texture and semantic integrated small objects detection in foggy scenes”.

**Point 2:** In the abstract, the conclusion of ‘the existing small target detection algorithms ignore the texture information’ is not very precise. U can correct it with ‘some of the existing small target detection algorithms’.

**Response:** There is something wrong with the content description. It should be “the existing small target detection algorithms ignore the texture information of tiny objects”. We modified it.

**Point 3:** The abstract should be rewritten carefully; then I could find how your experiment arranged logically.

**Response:** We have improved the abstract.

**Point 4:** Self-attention mechanism is applied in semantic information enhancement, and it is a very important part in this manuscript so I think it should be added in keywords.

**Response:** “Self-attention mechanism” is a keyword, because the PLOS ONE's template does not reflect the keywords, it is not reflected in this manuscript.

**Point 5:** In lines 134-136, I recommend to add citations supporting the statement of self-attention. Because it is not the conclusion of the article itself, so it should be expressed clearly.

**Response:** There is a corresponding introduction in the cited reference [19]( Hu. H, Gu. J, Zhang. Z, Dai. J and Wei. Y. Relation Networks for Object Detection[C]. IEEE Conference on Computer Vision and Pattern Recognition (CVPR), Salt Lake City, USA, 2018:3588-3597.). Therefore, we do not specifically cite reference.

**Point 6:** According to the foggy images, have u used any method to defog these images? If u have had some experiments, please show the results in the manuscript. If not, u can defogging these images, and they can be input to ur proposed algorithm. If u don’t think it necessary, please tell me why.

**Response:** In the revision manuscript, we supplemented the ablation experiments combined with different modules in experiments section.
